# Skeletal Muscle Adaptations to Exercise Training in Young and Aged Horses

**DOI:** 10.3389/fragi.2021.708918

**Published:** 2021-10-27

**Authors:** Christine M. Latham, Randi N. Owen, Emily C. Dickson, Chloey P. Guy, Sarah H. White-Springer

**Affiliations:** Texas A&M AgriLife Research and Department of Animal Science, Texas A&M University, College Station, TX, United States

**Keywords:** aging, exercise, horse, mitochondria, satellite cell, fiber type

## Abstract

In aged humans, low-intensity exercise increases mitochondrial density, function and oxidative capacity, decreases the prevalence of hybrid fibers, and increases lean muscle mass, but these adaptations have not been studied in aged horses. Effects of age and exercise training on muscle fiber type and size, satellite cell abundance, and mitochondrial volume density (citrate synthase activity; CS), function (cytochrome *c* oxidase activity; CCO), and integrative (per mg tissue) and intrinsic (per unit CS) oxidative capacities were evaluated in skeletal muscle from aged (*n* = 9; 22 ± 5 yr) and yearling (*n* = 8; 9.7 ± 0.7 mo) horses. Muscle was collected from the gluteus medius (GM) and triceps brachii at wk 0, 8, and 12 of exercise training. Data were analyzed using linear models with age, training, muscle, and all interactions as fixed effects. At wk 0, aged horses exhibited a lower percentage of type IIx (*p* = 0.0006) and greater percentage of hybrid IIa/x fibers (*p* = 0.002) in the GM, less satellite cells per type II fiber (*p* = 0.03), lesser integrative and intrinsic (*p* ≤ 0.04) CCO activities, lesser integrative oxidative phosphorylation capacity with complex I (P_CI_; *p* = 0.02) and maximal electron transfer system capacity (E_CI+II_; *p* = 0.06), and greater intrinsic P_CI_, E_CI+II_, and electron transfer system capacity with complex II (E_CII_; *p* ≤ 0.05) than young horses. The percentage of type IIx fibers increased (*p* < 0.0001) and of type IIa/x fibers decreased (*p* = 0.001) in the GM, and the number of satellite cells per type II fiber increased (*p* = 0.0006) in aged horses following exercise training. Conversely, the percentage of type IIa/x fibers increased (*p* ≤ 0.01) and of type IIx fibers decreased (*p* ≤ 0.002) in young horses. Integrative maximal oxidative capacity (*p* ≤ 0.02), E_CI+II_ (*p* ≤ 0.07), and E_CII_ (*p* = 0.0003) increased for both age groups from wk 0 to 12. Following exercise training, aged horses had a greater percentage of IIx (*p* ≤ 0.002) and lesser percentage of IIa/x fibers (*p* ≤ 0.07), and more satellite cells per type II fiber (*p* = 0.08) than young horses, but sustained lesser integrative and intrinsic CCO activities (*p* ≤ 0.04) and greater intrinsic P_CI_, E_CI+II_, and E_CII_ (*p* ≤ 0.05). Exercise improved mitochondrial measures in young and aged horses; however, aged horses showed impaired mitochondrial function and differences in adaptation to exercise training.

## Introduction

The three primary myosin heavy chain isoforms in horses are types I, IIa, and IIx (Rivero et al., 1996b), which are most commonly phenotypically characterized by differences in twitch speed and oxidative capacity (Table 1), but also often differ in capillarity, fatigability, and other variables. Individual muscle fibers can be composed of a single MyHC isoform in pure fibers, or of a mix of isoforms in hybrid fibers. Muscle fibers therefore span a spectrum of twitch speed, size, and oxidative capacity (Rivero et al., 1996a). Research in humans has demonstrated that exercise offers many benefits to skeletal muscle health, including increases in force production, muscle fiber size, and the percentage of fast, fatigue-resistant type IIa fibers (Williams et al., 2002). Improvements in these parameters are, in part, attributed to an increase in satellite cell number and activity with exercise training (Abreu et al., 2017). In agreement with the increase in size and percentage of fatigue-resistant oxidative fibers, exercise increases skeletal muscle oxidative capacity through a number of mechanisms including increases in mitochondrial biogenesis and fusion (Hood et al., 2019).

**TABLE 1:** General characteristics of muscle fiber types in normal, healthy muscle of horses.

	MyHC Type I	MyHC Type IIa	MyHC Type IIx
Twitch speed	Slow	Fast	Fast
Oxidative capacity	High	High	Low
Mitochondrial density	High	High	Low
Fatigue rate	Slow	Intermediate	Fast
Capillary density	High	Intermediate	Low
Force production	Low	Intermediate	High
Force velocity	Slow	Intermediate	Fast

Many translational studies of aging and exercise employ rodent models, which have some limitations in translation to human exercise and aging. Namely, human skeletal muscle does not contain myosin heavy chain (MyHC) type IIb fibers (Bloemberg and Quadrilatero, 2012), which can make drawing conclusions about sarcopenia particularly difficult in light of the fact that type II fibers are greatly impacted by age-related muscle wasting (Aagaard et al., 2010). Additionally, the lifespan of horses (approximately 25–30 yr) is closer to humans than the lifespan of rodents (approximately 2 yr). Thus, horses have the potential to provide a more translationally relevant model over the course of their lifespan. Furthermore, horses have the advantage of being a relatively large species, where skeletal muscle samples can be taken at multiple time points surrounding interventions, eliminating the necessity of euthanasia to obtain skeletal muscle data. Taken together, horses provide a promising model of aging, and their study has the potential to improve the health and wellbeing of both aging humans and horses. Previous work in horses has reported similar adaptations to aging as those seen in humans: decreased skeletal muscle mitochondrial number (Li et al., 2016), elevated muscular and circulating levels of inflammatory cytokines (McFarlane and Holbrook, 2008), and decreased muscle mass (Hintz, 2021). However, other parameters such as the prevalence of hybrid fibers, or those skeletal muscle fibers co-expressing multiple myosin heavy chain isotypes, have yet to be examined in horses.

A number of interventions have been utilized to improve age-related alterations in skeletal muscle parameters individually, including dietary antioxidant supplementation (Fusco et al., 2007) and anti-inflammatory interventions (Lambert et al., 2008; Elhassan et al., 2019), but these do not address the collective global alterations associated with aging. Low-intensity exercise has the potential to improve mitochondrial density, function and oxidative capacity, decrease the prevalence of hybrid fibers, and increase lean muscle mass. While the observed increases in oxidative capacity and lean muscle mass are intuitively beneficial, the decrease in prevalence of hybrid fibers may also be favorable, as an increase in the presence of hybrid fibers has been noted in both aging and disuse atrophy (Canepari et al., 2010), and may indicate the presence of damaged and denervated muscle fibers (Adams, 2006; Purves-Smith et al., 2014). However, the effect of exercise on these parameters in aged horses has not been closely studied. The objective of this study was to characterize the effects of age and exercise training on skeletal muscle fiber type, satellite cell abundance, and mitochondrial density, function, and oxidative capacity. We hypothesized that aged horses would have suppressed satellite cell abundance and mitochondrial measures, and alterations in muscle fiber type when compared to young horses, and that these parameters would be improved following 12 wk of light exercise training.

## Materials and Methods

### Horses

This study was reviewed and approved by the Texas A&M Institutional Animal Care and Use Committee (IACUC 2016-0294). Ten aged (nine mares and one gelding; 22 ± 4.5 yr) and eight young (three fillies and five colts; 9.7 ± 0.7 mo) Quarter Horses were used in this study. Horses were housed in paddocks (0.53, 0.72, and 0.60 ha for young colts, young fillies, and aged horses, respectively) devoid of fresh grass at the Texas A&M University Freeman Equestrian Center in College Station, TX. Colts were not yet castrated, and were therefore housed separately from fillies.

### Diets

Horses were allocated to separate pens by the group described above and had *ad libitum* access to Coastal bermudagrass hay. Hay intake per horse per day was estimated by the following formula:

$$\frac{Total\;hay\;offered\;in\;the\;pen\;per\;day\;\left(kg\right)-total\;hay\;refused\;in\;the\;pen\;per\;day\;\left(kg\right)}{Number\;of\;horses\;in\;the\;pen}$$

To meet nutrient requirements for each separate age group of horses (National Research Council, 2007), concentrate grain was offered to aged horses at 0.5% body weight (BW)/d [dry matter (DM) basis] and to young horses at 1.25% BW/d (DM basis). Horses received grain meals individually in stalls (3.7 × 3.7 m) split equally into two meals fed at 0700 h and 1700 h. Refusals were monitored and recorded daily to calculate actual concentrate intake. Diets were formulated to maintain a body condition score (BCS) of 5–6 according to the Henneke body condition scoring system (Henneke et al., 1983), and to meet all requirements of mature or growing horses in light exercise, as appropriate (National Research Council, 2007). Throughout the study, BW of horses was recorded weekly using a livestock scale accurate to 1 kg (Cardinal Scales, Webb City, MO, United States).

### Exercise

Horses had received no forced exercise for at least 6 mo prior to the beginning of this study. Beginning at wk 0, horses were enrolled in a 12-wk submaximal exercise training program. Exercise was designed to achieve light work as defined by the NRC (National Research Council, 2007), and consisted of 12 min of walking, 15 min of trotting, and 3 min of cantering in a free-stall exerciser (30 min total/day) 5 days/wk. Each gait was performed in both directions each day, and horses alternated starting the exercise bout clockwise or counterclockwise each day. For aged horses, the walk was performed at a speed of 1.2 m/s, the trot at 2.5 m/s, and the canter at 5.2 m/s. Owing to changes in stride length with growth, the speed of each gait for young horses was progressively increased throughout the study to ensure that all horses remained in the intended gait. Gait speeds for young horses started at 1.1 m/s for the walk, 2.5 m/s at the trot, and 5.0 m/s at the canter at the beginning of training and were increased to 1.2 m/s at the walk, 3.0 m/s at the trot, and 5.4 m/s at the canter by the end of the 12-wk exercise training program.

### Sample Collection

Skeletal muscle samples were collected at wk 0, 8, and 12, for analysis of fiber type, satellite cell abundance, citrate synthase (CS) and cytochrome *c* oxidase (CCO) activities, and complex-specific oxidative and electron transfer capacities. Samples were collected from the gluteus medius (GM) and triceps brachii (TB) using a tissue collection procedure as previously described (White et al., 2016). These muscle groups were selected to represent two different muscle group functions and fiber type distributions. The GM is a propulsive muscle group, with a greater proportion of fast twitch nonoxidative fibers, whereas the TB is used more for stabilization, and therefore has a more slow-twitch oxidative muscle fiber type distribution (Van den Hoven et al., 1985; Kawai et al., 2009). Horses were sedated with detomidine hydrochloride (Dormosedan; Zoetis, Parsippany-Troy Hills, NJ, United States) prior to beginning tissue collection procedures. The collection areas were clipped, scrubbed with a 7.5% povidone-iodine solution, and then rinsed with a 70% ethanol solution. The tissue collection sites were desensitized with 0.5 ml of 2% lidocaine (Vetone, Boise, ID) and a 14-gauge needle was used to create the initial puncture through the skin. Tissue was collected using a 14-gauge, 9-cm tissue collection needle (SuperCore; Argon Medical Devices Inc., Frisco, TX, United States) inserted to a depth of 5 cm in aged horses and 3.5 cm in young horses. The tissue collection site alternated between the left and right sides of the horse at each sampling interval. Samples obtained from the same side of the horse were collected approximately 2 cm from the previous insertion site. At each sampling interval, approximately 300 mg (wet weight) of muscle tissue was flash frozen in liquid nitrogen and stored at −80 C until enzyme activity analyses were performed. For muscle fiber type analysis, approximately 400 mg tissue was embedded in an optimal cutting temperature (OCT) compound (Fisher Scientific, Hampton, NH, United States), frozen in liquid nitrogen-cooled isopentane, and stored at −80°C until analysis. For high-resolution respirometry (HRR), muscle fibers were collected into an ice-cold biopsy preservation medium (BIOPS) (Fontana-Ayoub et al., 2014) and stored on ice or at 4°C until analysis.

### Enzyme Activities

Flash frozen muscle was cryopulverized into a fine powder (Spectrum™ Bessman Tissue Pulverizer; Thermo Fisher Scientific, Waltham, MA, United States) for the evaluation of CS and CCO activities as markers of mitochondrial volume density and function, respectively (Larsen et al., 2012; Meinild Lundby et al., 2018). Samples were prepared and activities were measured as previously described (Spinazzi et al., 2012; Li et al., 2016). Briefly, CS activity was assessed at 412 nm by measuring the initial rate of reaction of free CoA-SH with DTNB; CCO activity was determined by measuring the maximal, linear rate of oxidation of fully reduced cytochrome *c* at 550 nm. Enzymatic activities were normalized to homogenate supernatant protein content, determined using the Bradford Protein Assay Kit (Thermo Fisher Scientific). Cytochrome *c* oxidase activity is presented on an integrative (per mg protein) and intrinsic (per unit CS) basis.

### Immunohistochemistry

For all immunohistochemistry, muscle tissue samples were cut into 7 µm sections using a cryostat (Leica Biosystems, Wetzlar, Germany), air dried onto glass slides for 1 h (Fisher Scientific) and stored at −20°C until analysis. Before analysis, sections were separated with a hydrophobic pen (Vector Laboratories, Burlingame, CA, United States) and rehydrated with 0.01 M phosphate-buffered saline (PBS; Fisher Scientific).

To determine muscle fiber type, antibodies for myosin heavy chain (MyHC) type I (BA-D5), type IIa (SC-71), and type IIx (6H1; all from Developmental Studies Hybridoma Bank (DSHB), University of Iowa) were used (Schiaffino et al., 1989; Barrey et al., 1998; Rivero et al., 1999; Votion et al., 2007; Latham and White, 2017). Briefly, sections were incubated concurrently in BA-D5 (1:100), SC-71 (1:100), and 6H1 (undiluted supernatant) for 90 min at room temperature (RT). Sections were washed and incubated concurrently in the appropriate fluorescent secondary antibody (goat anti-mouse IgG2b Alexa Fluor 647 (type I; 1:250), goat anti-mouse IgG1 Alexa Fluor 488 (type IIa; 1:500), and goat anti-mouse IgM Alexa Fluor 555 (type IIx; 1:250), Thermo Fisher Scientific) diluted in PBS.

**FIGURE 1:**
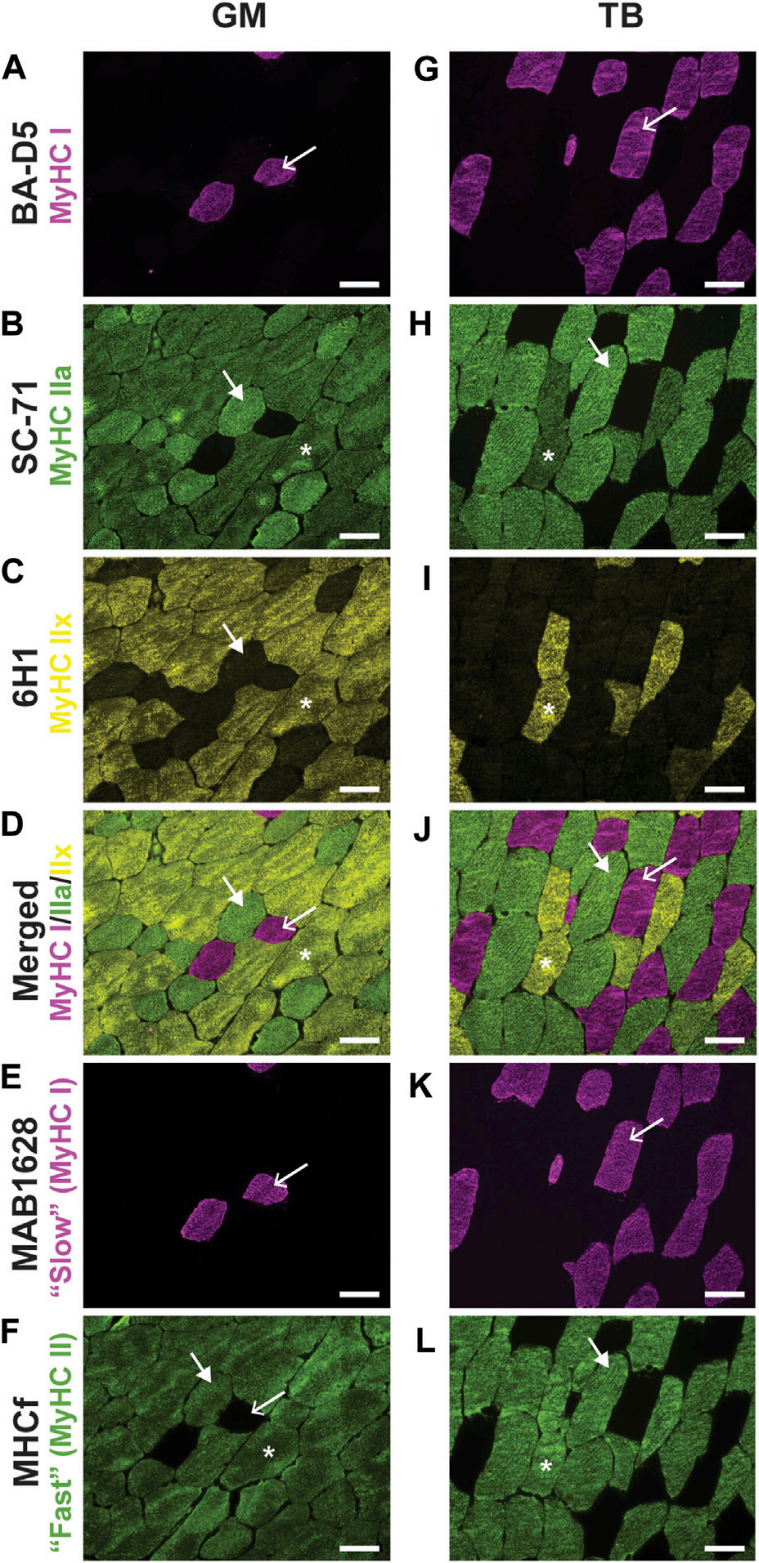
Representative images of fluorescent staining of horse gluteus medius (GM) and triceps brachii (TB) myosin heavy chain (MyHC) type I using primary antibodies BA-D5 **(A**,**G)** and MAB1628 **(E**,**K)**, MyHC type IIa using the primary antibody SC-71 **(B**,**H)**, MyHC type IIx using the primary antibody 6H1 **(C**,**I)**, a merged image of concurrently stained BA-D5, SC-71, and 6H1 **(D**,**J)**, and MyHC type IIa and IIx using the primary antibody MHCf **(F**,**L)**. Within a column, arrows point to the same fiber. Arrow, MyHC type I (pink); large arrowhead, MyHC IIa (green); asterisks indicate type IIa/x hybrid fibers. No pure MyHC IIx (yellow) fibers were noted in these sections. Scale bar, 100 µm.

To confirm proper staining of types I, IIa, and IIx, a small preliminary experiment was performed, in which sections stained with the above antibodies from DSHB were compared to serial sections incubated in previously validated primary antibodies in horses: *1*) primary antibody for slow twitch fibers (MAB1628; Millipore Sigma, Burlington, MA, United States) (Tulloch et al., 2011); and *2*) primary antibody for “fast” fibers (assumed to be MyHC type II; MHCf; Leica Biosystems). The myosin heavy chain type I antibody, BA-D5 (Figures 1A,G), and the antibody against slow twitch fibers, MAB1628 (Figures 1E,K) identified the same fibers for all type I fibers counted. Together, 6H1 and SC-71 (Figures 1B,C,H,I) labeled the same fibers as MHCf (Figures 1F,L). This preliminary work validated the use of DSHB primary antibodies to detect MyHC types I, IIa, and IIx in equine skeletal muscle.

To determine satellite cell (SC) abundance, sections were probed with a Pax7 antibody previously used in horses (Kawai et al., 2013). Sections were fixed in ice-cold acetone for 3 min at −20°C to preserve the genomic antigen Pax7. Endogenous peroxidases were blocked in 3% hydrogen peroxide solution for 7 min at RT to prevent the production of background signal during the development of the horseradish peroxidase signal, and then sections were blocked with 10% normal goat serum at RT for 1 h. Sections were incubated overnight at 4°C in Pax7 (1:100; DSHB) and laminin (1:300; Millipore Sigma). On the next day, sections were incubated for 1 h in goat anti-mouse biotinylated secondary antibody (1:1,000; Thermo Fisher Scientific) and goat anti-rabbit IgG Alexa Fluor 594 (1:1,000; Thermo Fisher Scientific) diluted in 10% normal goat serum. Sections were incubated in streptavidin-horseradish peroxidase (Thermo Fisher Scientific) for 1 h at RT, and then Pax7 signal was developed with an Alexa Fluor 488 tyramide signal amplification kit (Thermo Fisher Scientific) per manufacturer’s instructions. Sections were then incubated for 90 min at RT in BA-D5 diluted in PBS (1:100), followed by a 1 h incubation at RT in goat anti-mouse IgG2b Alexa Fluor 647 (Thermo Fisher Scientific) diluted in PBS (1:250).

Slides were mounted with fluorescent mounting media (Vector Laboratories, Burlingame, CA, United States) and stored at 4°C until imaging. For Pax7 abundance, slides were mounted with fluorescent mounting media containing DAPI (Vector Laboratories). Sections were imaged with the appropriate fluorescent filter for immunohistochemistry (Nikon Instruments, Melville, NY, United States). A minimum of 50 muscle fibers were compared for each sample. Fiber type-specific minimum feret diameter was analyzed as an indicator of muscle fiber size using ImageJ (National Institutes of Health, Bethesda, MD, United States). The number of satellite cells per type I fiber was calculated by dividing the number of satellite cells present in the laminin border of type I fibers by the total number of type I fibers counted for each sample. Similarly, the number of satellite cells per type II fiber was calculated by dividing the number of satellite cells present in the laminin border of type II fibers by the total number of type II fibers counted for each sample. The percentage of Pax7-positive nuclei was calculated as the number of Pax7-positive nuclei counted divided by the total number of nuclei counted for each sample, multiplied by 100.

### High-Resolution Respirometry

For high-resolution respirometry (HRR), muscle fibers were collected, prepared, and permeabilized as previously described (Li et al., 2016) and then analyzed within 24 h of collection. Oxygen flux and respiratory states were determined by HRR using an Oxygraph-2k respirometer (Oroboros, Innsbruck, Austria) with the following substrate-uncoupler-inhibitor titration protocol (Supplementary Figure 1) previously described for equine skeletal muscle (Latham et al., 2019): *1*) pyruvate (5 mM) and malate (1 mM) to support electron flow through complex I (CI) of the electron transfer system (ETS; LEAK respiration); *2*) adenosine diphosphate (ADP; 2.5 mM) to stimulate respiration (OXPHOS) and cytochrome *c* (cyt c; 10 μM) to assess outer mitochondrial membrane integrity (samples with responses to cyt c greater than 15% were reanalyzed); *3*) glutamate (10 mM) as an additional CI substrate to assess OXPHOS with complex I (P_CI_); *4*) succinate (10 mM) to support convergent electron flow through complex II (CII) of the ETS (P_CI+II_); *5*) uncoupler carbonyl cyanide 3-chlorophenylhydrazone (CCCP; 0.5 μM steps) to assess maximal noncoupled ETS capacity (E_CI+II_; noncoupled maximal respiration indicates the capacity of the ETS when it is not constrained by the rate at which ATP synthase can restore the hydrogen ion gradient); *6*) rotenone (0.5 μM), an inhibitor of complex I, to measure ETS capacity of complex II alone (E_CII_); *7*) antimycin A (2.5 μM), an inhibitor of complex III, to measure residual oxygen flux (ROX) independent of the ETS. Data were collected using DatLab software (version 7.0, Oroboros). HRR data are presented as integrative (relative to tissue wet weight) and intrinsic (relative to U CS) capacities. Sample flux control ratio (FCR) for each complex was calculated by dividing the flux in each complex by the sample’s E_CI+II_ flux.

### Statistical Analysis

Differences in muscle fiber type, satellite cell abundance, enzyme activities, and mitochondrial respiration measurements were analyzed using the MIXED procedure of SAS 9.4 (SAS Institute, Inc, Cary, NC, United States) with repeated measures. Age (aged or young), training (wk 0, 8, and 12), muscle group (GM and TB), and all interactions were included in the model as fixed effects. Though muscle group and its interactions were included in the model, data for the GM are graphed in separate panels from the TB to allow for clearer representation of the data. Sex was initially included in the models as a fixed effect but removed due to lack of significance (*p* > 0.1) All data are expressed as least squared means ± SEM. Significance was considered at *p* ≤ 0.05, and trends were acknowledged at 0.05 ≤ *p* ≤ 0.10.

## Results

### Muscle Fiber Type and Size

#### Age and Training

**FIGURE 2:**
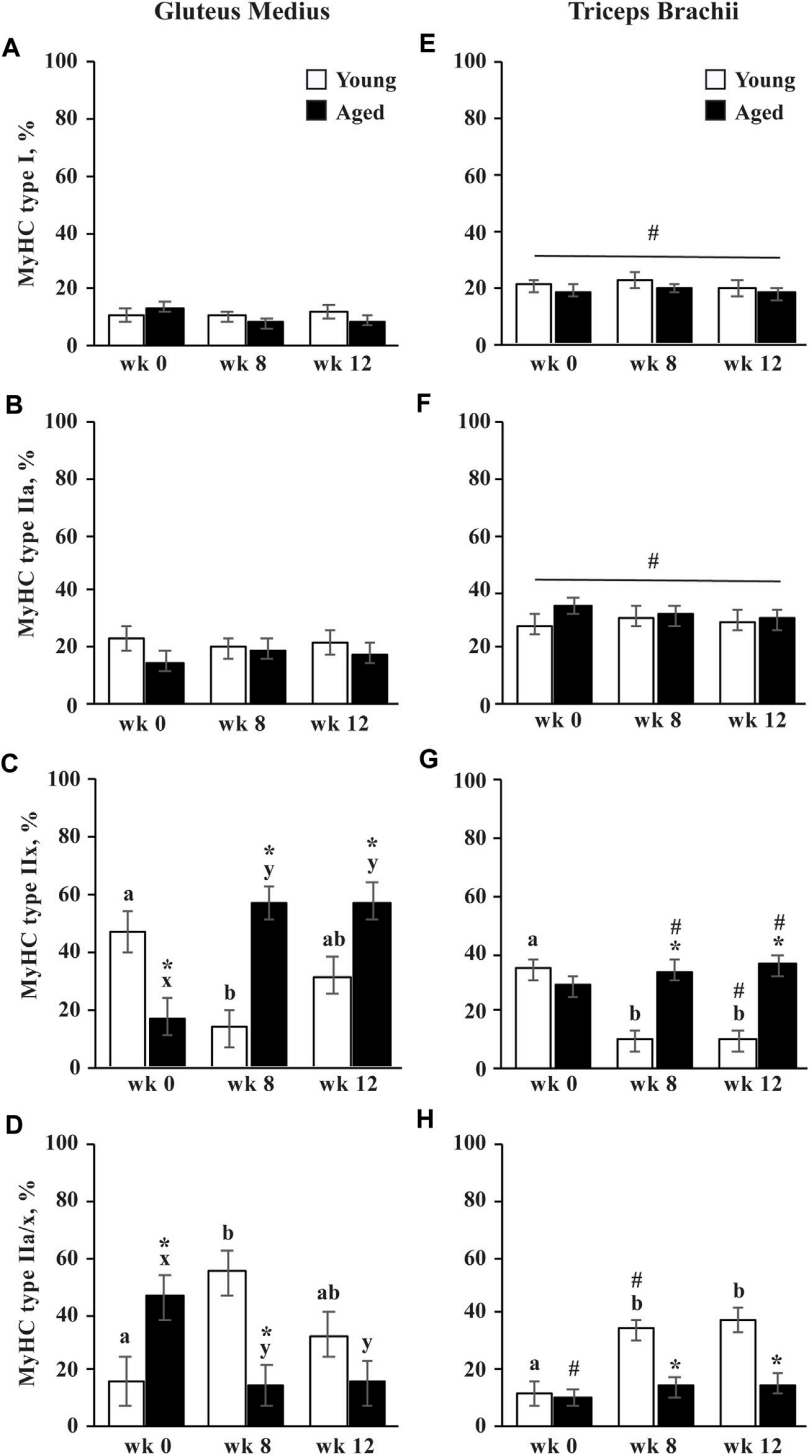
Gluteus medius and triceps brachii myosin heavy chain (MyHC) type I **(A**,**E)**, type IIa **(B**,**F)**, type IIx **(C**,**G)**, and hybrid type IIa/x **(D**,**H)** percentage before (wk 0), and after 8 (wk 8) and 12 (wk 12) weeks of submaximal exercise training in aged (22 ± 4.5 yr; *n* = 10) and young (9.7 ± 0.7 mo; *n* = 8) horses. Overall effect of age (*p* = 0.3; *p* = 0.8; *p* = 0.0005; *p* = 0.02), training (*p* = 0.7; *p* = 0.99; *p* = 0.4; *p* = 0.12), muscle group (*p* < 0.0001; *p* < 0.0001; *p* = 0.001; *p* = 0.01), age × training (*p* = 0.5; *p* = 0.9; *p* < 0.0001; *p* < 0.0001), age × muscle group (*p* = 0.6; *p* = 0.12; *p* = 0.8; *p* = 0.4), training × muscle group (*p* = 0.32; *p* = 0.9; *p* = 0.02; *p* = 0.03), and age × training × muscle group (*p* = 0.5; *p* = 0.3; *p* = 0.02; *p* = 0.01) for MyHC type I, type IIa, type IIx, and type IIa/x, respectively. * Within a muscle group and week, aged differs from young (*p* < 0.05). ^#^ Within a week and age group, GM differs from TB (*p* < 0.05). ^a,b^ Within young horses and muscle group, time points with different letters differ (*p* < 0.05). ^x,y^ Within aged horses and muscle group, time points with different letters differ (*p* < 0.05).

The percentage of type I and type IIa did not change in response to training and were not different between age groups. Throughout training, type IIa fibers were larger in aged than young horses (*p* = 0.002; Table 1). Before exercise training began, the GM of aged horses had a lesser percentage of type IIx fibers than young horses (*p* = 0.0006; Figure 2C). From wk 0 to 8, the percentage of type IIx fibers increased (*p* < 0.0001) in aged horse GM and remained greater in aged horses through wk 12 compared to wk 0 (*p* < 0.0001). The percentage of type IIx fibers did not change throughout the study in the TB of aged horses (Figure 2G). Conversely, the percentage of type IIx fibers decreased in young horses from wk 0 to 8 in both muscle groups (*p* ≤ 0.002 Figures 2C,G) and remained lower than wk 0 (GM, *p* = 0.06; TB, *p* = 0.002) through wk 12 in young horses. Owing to differences in adaptation to exercise, aged horses had a greater percentage of type IIx fibers at wk 8 and 12 compared to young horses (*p* ≤ 0.002). Throughout training, type IIx fibers tended to be larger (*p* = 0.07) in aged than young horses (Table 2). A trend for an effect of training (*p* = 0.09) suggested that type IIx fibers decreased in size from wk 0 to 12 (*p* = 0.03) in both the age groups. While the size of type IIa/x fibers did not change in young horses, they decreased in size in aged horses from wk 0 to 12 (*p* = 0.0008; Table 2). Because of these differences in adaptations between young and aged horses, type IIa/x fibers were larger in aged than young horses through wk 8 (*p* ≤ 0.04) but were not different between age groups at wk 12. A trend for a training × muscle group interaction (*p* = 0.07) suggested that type IIa/x fibers in the GM were smaller at wk 0 than at wk 8 or 12 (*p* ≤ 0.03), but their size did not change with training in the TB.

**TABLE 2:** Minimum feret diameter (A.U.) of gluteus medius (GM) and triceps brachii (TB) myosin heavy chain (MyHC) type I, type IIa, type IIx, and hybrid type IIa/x before (wk 0) and after 8 (wk 8) and 12 (wk 12) weeks of submaximal exercise training in aged (22 ± 5 yr; *n* = 10) and young (9.7 ± 0.7 mo; *n* = 8) horses.

						*p*-values			
		wk 0	wk 8	wk 14	SEM	Age	Training	Muscle Group	Age × Training × Muscle Group
*MyHC type I*									
Young	GM	103	115	103	8	0.144	0.905	0.094	0.501
	TB	115	113	114					
Aged	GM	120	114	117					
	TB	118	123	128					
*MyHC type IIa*									
Young	GM	108	124	109	8	0.002	0.689	0.0001	0.395
	TB	135^a^	123^a^	132^a^					
Aged	GM	138^b^	128^b^	124^b^					
	TB	155^a^ ^,^ ^b^	158^a^ ^,^ ^b^	156^a^ ^,^ ^b^					
*MyHC type IIx*									
Young	GM	162	181	154	11	0.068	0.093	0.651	0.824
	TB	166	159	156					
Aged	GM	173	194	173					
	TB	183	187	176					
*MyHC type IIa/x*									
Young	GM	143	155	143	10	0.013	0.117	0.219	0.268
	TB	145	140	159					
Aged	GM	192^a^ ^,^ ^c^	162^a^ ^,^ ^c^	134^d^					
	TB	177^a^ ^,^ ^c^	178^a^ ^,^ ^c^	165^d^					

a Within a muscle group and time point, aged differs from young (p < 0.05).

b Within a week and age group, GM differs from TB (p < 0.05).

c Within an age and muscle group, time points with different letters differ (p < 0.05).

#### Muscle Group

The percentage of type I and type IIa fibers was greater in the TB than the GM (*p* < 0.0001; Figures 2A,B). For both the age groups, type I fibers tended to be larger (*p* = 0.09) and type IIa fibers were larger (*p* = 0.0001) in the TB than the GM (Table 2). Before exercise training, the percentage of type IIx fibers tended to be greater in the TB than the GM in aged horses (*p =* 0.10) but was not different between muscle groups in young horses (Figures 2C,G). The percentage of type IIx fibers was greater in the GM than the TB for aged horses by wk 8 (*p* = 0.0002) and for young horses by wk 12 (*p* = 0.007; Figures 2C,G). Before exercise training, the percentage of type IIa/x fibers was greater in the GM than the TB in aged horses (*p <* 0.0001) and was not different between muscle groups in young horses (Figures 2D,H). At wk 8, young horses had a greater percentage of IIa/x fibers in the GM than the TB (*p* = 0.02), but there was no difference between muscle groups for either age group by wk 12.

### Satellite Cell Abundance

**FIGURE 3:**
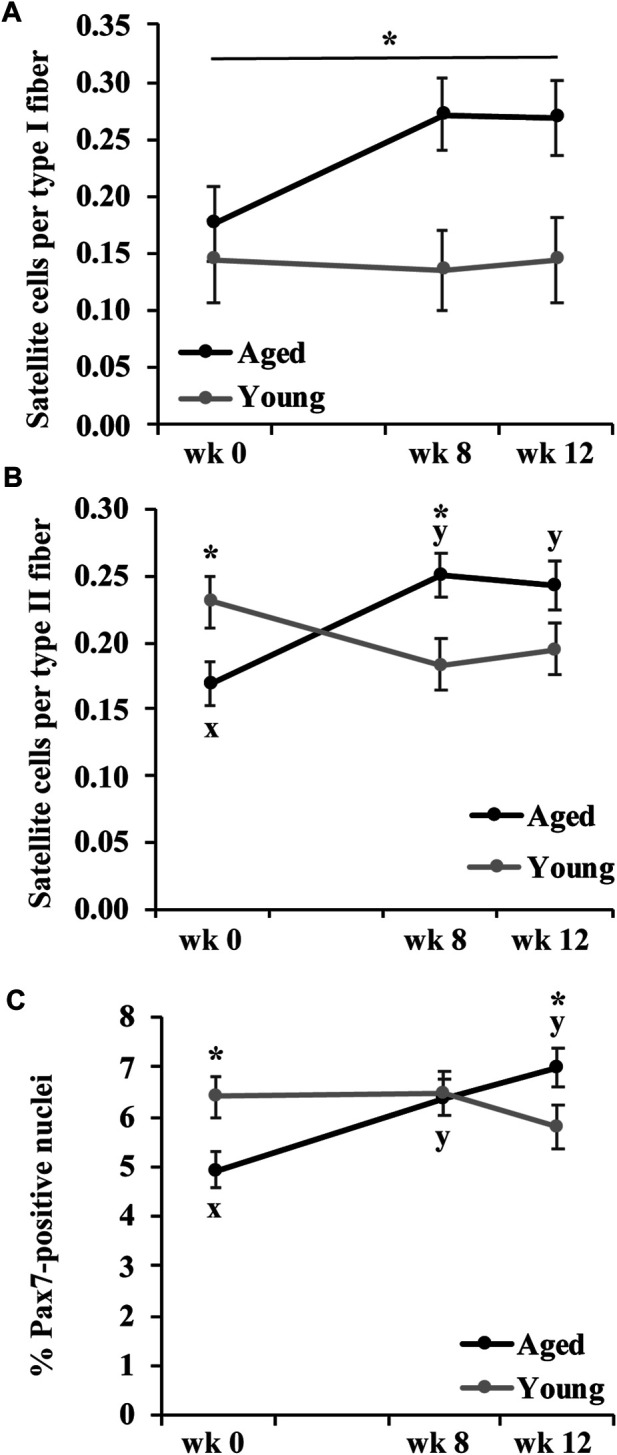
Gluteus medius and triceps brachii satellite cells per type I fiber **(A)**, satellite cells per type II fiber **(B),** and percent of Pax7-positive nuclei **(C)** before (wk 0), and after 8 (wk 8) and 12 (wk 12) weeks of submaximal exercise training in aged (22 ± 4.5 yr; *n* = 10) and young (9.7 ± 0.7 mo; *n* = 8) horses. Owing to lack of effect of muscle group, muscle groups have been combined. Overall effect of age (*p* = 0.0009; *p* = 0.3; *p* = 0.8), training (*p* = 0.3; *p* = 0.4; *p* = 0.056), muscle group (*p* = 0.1; *p* = 0.6; *p* = 0.8), age × training (*p* = 0.2; *p* = 0.0007; *p* = 0.003), age × muscle group (*p* = 0.9; *p* = 0.9; *p* = 0.6), training × muscle group (*p* = 0.96; *p* = 0.6; *p* = 0.4), and age × training × muscle group (*p* = 0.14; *p* = 0.6; *p* = 0.5) for satellite cells per type I fiber, satellite cells per type II fiber, and percent of Pax7-positive nuclei, respectively. * Within time point, aged differs from young (*p* < 0.05). ^x,y^ Within aged horses, time points with different letters differ (*p* < 0.05).

None of the measures of satellite cell abundance differed by muscle group so muscle groups were combined. The number of satellite cells per type I fiber was greater in aged than young horses (*p* = 0.009), but did not change in response to training (Figure 3A). The number of satellite cells per type II fiber (*p* = 0.03) was lower in aged than young horses before exercise training (Figure 3B). The number of satellite cells per type II fiber increased in aged horses (*p* = 0.0006), but it tended to decrease (*p* = 0.06) in young horses at wk 8. At wk 12, the number of satellite cells per type II fiber remained greater than wk 0 in aged horses (*p* = 0.002) but was not different from wk 0 for young horses. These differences in adaptation to exercise training between age groups led to more satellite cells per type II fiber at wk 8 (*p* = 0.01), and a trend for more satellite cells per type II fiber at wk 12 (*p* = 0.08) for aged compared to young horses (Figure 3B).

The percentage of Pax7-positive nuclei (*p* = 0.02) was lesser in aged than young horses before exercise training (Figure 3C) but increased in aged horses by wk 8 and remained greater at wk 12 than at wk 0 (*p* ≤ 0.003). In young horses, training did not impact percentage of Pax7-positive nuclei. There was no difference in percentage of Pax7-positive nuclei between age groups by wk 8, and aged horses had a greater percentage of Pax7-positive nuclei than young horses by wk 12 (*p* = 0.05; Figure 3C).

### Enzymatic Activities

#### Age and Training

**FIGURE 4:**
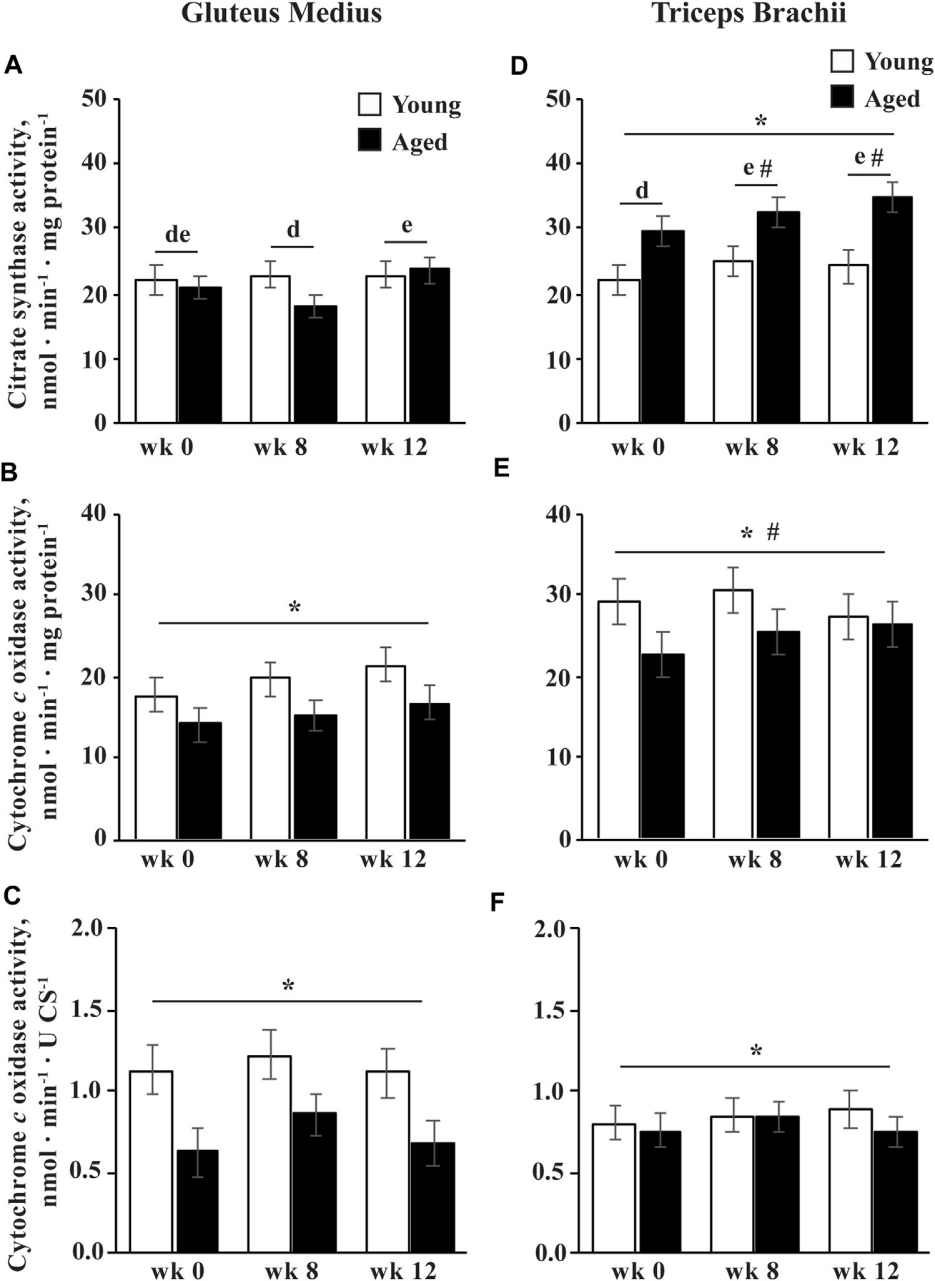
Gluteus medius and triceps brachii citrate synthase (CS) activity **(A**,**D)**, and integrative (per mg protein; **B**,**E**) and intrinsic (per unit CS; **C**,**F**) cytochrome *c* oxidase (CCO) activities before (wk 0), and after 8 (wk 8) and 12 (wk 12) weeks of submaximal exercise training in aged (22 ± 4.5 yr; *n* = 10) and young (9.7 ± 0.7 mo; *n* = 8) horses. Overall effect of age (*p* = 0.3; *p* = 0.04; *p* = 0.02), training (*p* = 0.01; *p* = 0.09; *p* = 0.13), muscle group (*p* = 0.0008; *p* < 0.0001; *p* = 0.93), age × training (*p* = 0.06; *p* = 0.3; *p* = 0.4), age × muscle group (*p* = 0.0007; *p* = 0.2; *p* = 0.14), training × muscle group (*p* = 0.02; *p* = 0.4; *p* = 0.6), and age × training × muscle group (*p* = 0.11; *p* = 0.96; *p* = 0.99) for CS activity, integrative CCO activity, and intrinsic CCO activity, respectively. * Within muscle group, aged differs from young (*p* < 0.05). ^#^ Within week, GM differs from TB (*p* < 0.05). ^d,e^ Within muscle group, time points with different letters differ (*p* < 0.05).

Citrate synthase activity was greater in aged than young horses in the TB (*p* = 0.004; Figure 4D). Integrative CCO activity was lower for aged than young horses (*p* = 0.04; Figures 4B,E). A trend for an effect of training (*p* = 0.09) indicated that integrative CCO activity tended to increase at wk 8 (*p* = 0.08) and increased significantly by wk 12 (*p* = 0.04) in both the age groups. Intrinsic CCO activity was also lower in aged than young horses (*p* = 0.02) but was not affected by training (Figures 4C,F).

#### Muscle Group

An age × muscle group interaction (*p* = 0.0007) indicated that CS activity was greater in the TB than the GM in aged (*p* < 0.0001), but not in young horses (Figures 4A,D). However, a training × muscle group interaction (*p* = 0.02) indicated that CS was not different between muscle groups at wk 0, but increased in the TB at wk 8 (*p* = 0.001) and remained greater at wk 12 (*p* = 0.003), leading to greater CS activity in the TB compared to the GM at wk 8 and 12 (*p* ≤ 0.03). Integrative CCO activity was higher in the TB than the GM (*p* < 0.0001; Figures 4B,E) but intrinsic CCO activity was not different between muscle groups (Figures 4C,F).

### Oxidative Capacities

#### Integrative Oxidative Capacities

**FIGURE 5:**
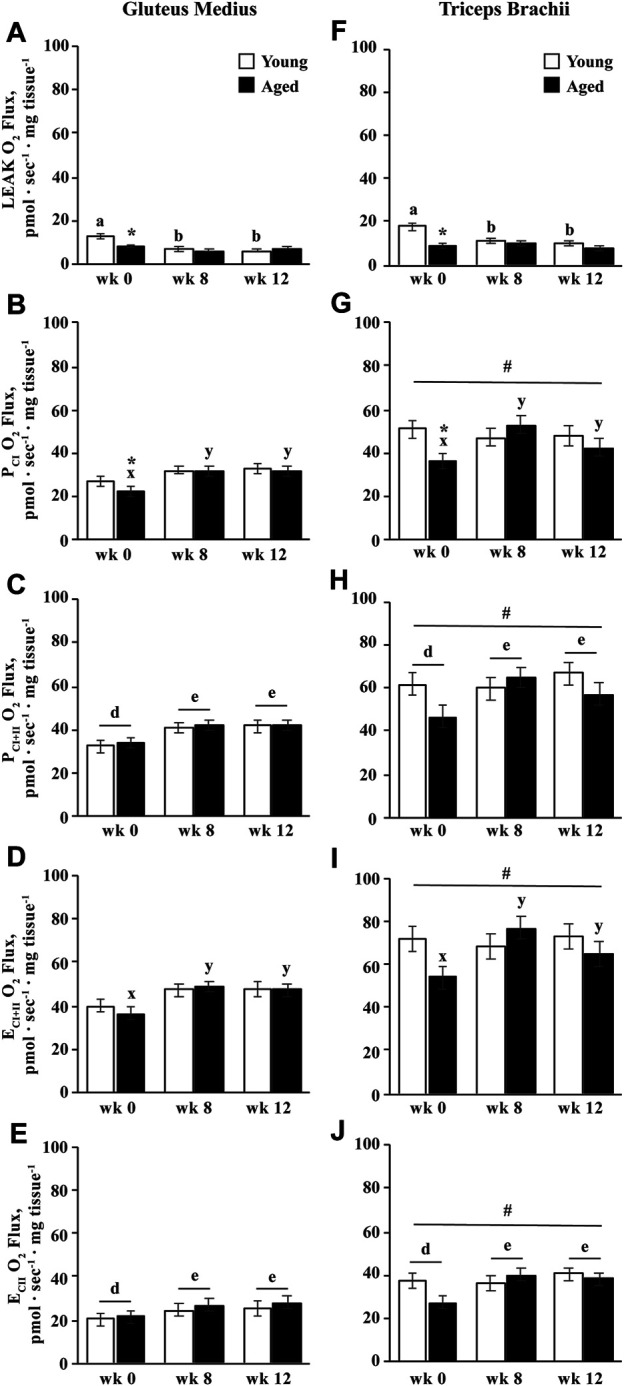
Integrative (mass-specific) mitochondrial respiration of permeabilized fibers from the gluteus medius and triceps brachii analyzed *via* high-resolution respirometry. LEAK respiration (LEAK; **A**,**F**), complex I-supported oxidative phosphorylation capacity (P_CI_; **B**,**G**), complex I and II-supported P (P_CI+II_; **C**,**H**), maximal noncoupled electron transfer system capacity (E_CI+II_; **D**,**I**), and complex II-supported E (E_CII_; **E**,**J**) were measured before (wk 0), and after 8 (wk 8) and 12 (wk 12) weeks of submaximal exercise training in aged (22 ± 4.5 yr; *n* = 10) and young (9.7 ± 0.7 mo; *n* = 8) horses. Overall effect of age (*p* = 0.0.002; *p* = 0.2; *p* = 0.2; *p* = 0.3; *p* = 0.6), training (*p* < 0.0001; *p* = 0.01; *p* = 0.003; *p* = 0.004; *p* = 0.0009), muscle group (*p* < 0.0001; *p* < 0.0001; *p* < 0.0001; *p* < 0.0001; *p* < 0.0001), age × training (*p* < 0.0001; *p* = 0.03; *p* = 0.10; *p* = 0.03; *p* = 0.13), age × muscle group (*p* = 0.01; *p* = 0.2; *p* = 0.2; *p* = 0.3; *p* = 0.2), training × muscle group (*p* = 0.13; *p* = 0.16; p = 0.6; *p* = 0.4; *p* = 0.8), and age × training × muscle group (*p* = 0.17; *p* = 0.19; *p* = 0.2; *p* = 0.2; *p* = 0.2) for LEAK, P_CI_, P_CI+II_, E_CI+II_, and E_CII_, respectively. * Within a muscle group and week, aged differs from young (*p* < 0.05). ^#^ GM differs from TB (*p* < 0.05). ^a,b^ Within young horses, time points with different letters differ (*p* < 0.05). ^x,y^ Within aged horses, time points with different letters differ (*p* < 0.05). ^d,e^ Within muscle group, time points with different letters differ (*p* < 0.05).

Integrative oxidative capacity is normalized to muscle tissue wet weight, and therefore represents oxidative capacity of the muscle as a whole, without accounting for differences or changes in mitochondrial volume density. An age × muscle group interaction (*p* = 0.01) indicated that integrative LEAK was greater for young than aged horses in the TB (*p* = 0.0003; Figure 5F). However, an age × training interaction showed that integrative LEAK respiration decreased by wk 8 in young horses (*p* < 0.0001) and remained lower at wk 12 (*p* < 0.0001). Thus, while integrative LEAK respiration was greater in young than aged horses before exercise training (*p* < 0.0001), there was no difference between age groups at wk 8 or 12 (Figures 5A,F).

Aged horses exhibited lower integrative P_CI_ than young horses before exercise training (*p* = 0.02; Figures 5B,G). Integrative P_CI_ increased by wk 8 in aged horses (*p* = 0.0001), and tended to decrease from wk 8 to 12 (*p* = 0.09). Conversely, integrative P_CI_ did not change with training in young horses, and there was therefore no difference between age groups by wk 8 (Figure 5B). Overall, integrative P_CI+II_ increased by wk 8 (*p* = 0.006) and remained greater than wk 0 at wk 12 (*p* = 0.001; Figures 5C,H). A trend for an age × training interaction (*p* = 0.10) suggested that while P_CI+II_ increased for aged horses by wk 8 (*p* = 0.001), young horses only tended to increase by wk 8 (*p* = 0.08). Both aged (*p* = 0.02) and young (*p* = 0.01) horses showed elevated P_CI+II_ at wk 12 compared to wk 0, but aged horses tended to have lower P_CI+II_ than young horses at wk 12 (*p* = 0.07; Figures 5C,H). Similarly, integrative E_CI+II_ increased in aged horses by wk 8 (*p* = 0.0002) and remained greater at wk 12 (*p* = 0.02), while young horses only tended to increase by wk 12 (*p* = 0.07; Figures 5D,I). Therefore, while E_CI+II_ tended to be lower in aged than young horses at wk 0 (*p* = 0.06), there was no difference between age groups at wk 8 or 12. Integrative E_CII_ increased by wk 8 (*p* = 0.004) and remained elevated at wk 12 (*p* = 0.0003), but did not differ between aged and young horses (Figures 5E,J). Integrative LEAK, P_CI_, P_CI+II_, E_CI+II_, and E_CII_ (*p* ≤ 0.02) were greater in the TB than the GM (Figure 5).

#### Intrinsic Oxidative Capacities

**FIGURE 6:**
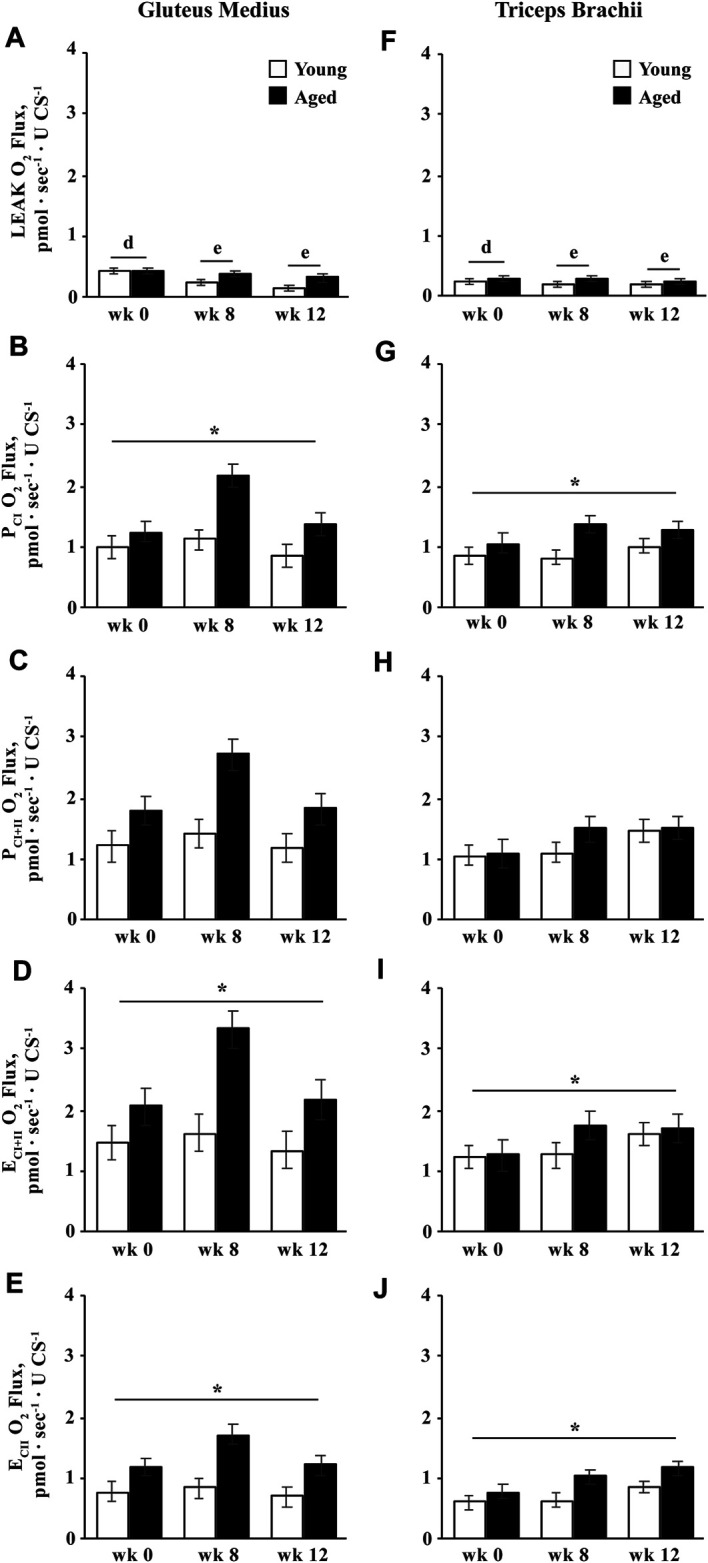
Intrinsic (normalized to CS activity) mitochondrial respiration of permeabilized fibers from the gluteus medius and triceps brachii analyzed *via* high-resolution respirometry. LEAK respiration (LEAK; **A**,**F**), complex I-supported oxidative phosphorylation capacity (P_CI_; **B**,**G**), complex I and II-supported P (P_CI+II_; **C**,**H**), maximal noncoupled electron transfer system capacity (E_CI+II_; **D**,**I**), and complex II-supported E (E_CII_; **E**,**J**) were measured before (wk 0), and after 8 (wk 8) and 12 (wk 12) weeks of submaximal exercise training in aged (22 ± 4.5 yr; *n* = 10) and young (9.7 ± 0.7 mo; *n* = 8) horses. Overall effect of age (*p* = 0.15; *p* = 0.05; *p* = 0.05; *p* = 0.04; *p* = 0.04), training (*p* = 0.0005; *p* = 0.056; *p* = 0.12; *p* = 0.10; *p* = 0.12), muscle group (*p* = 0.2; *p* = 0.5; *p* = 0.5; *p* = 0.3; *p* = 0.3), age × training (*p* = 0.06; *p* = 0.11; *p* = 0.2; *p* = 0.14; *p* = 0.2), age × muscle group (*p* = 0.7; *p* = 0.9; *p* = 0.99; *p* = 0.9; *p* = 0.8), training × muscle group (*p* = 0.3; *p* = 0.6; *p* = 0.5; *p* = 0.5; *p* = 0.4), and age × training × muscle group (*p* = 0.8; *p* = 0.9; *p* = 0.9; *p* = 0.9; *p* = 0.97) for LEAK, P_CI_, P_CI+II_, E_CI+II_, and E_CII_, respectively. * Aged differs from young (*p* < 0.05). ^d,e^ Time points with different letters differ (*p* < 0.05).

Intrinsic oxidative capacity is normalized to muscle tissue CS activity, and therefore represents oxidative capacity normalized to mitochondrial density. Overall, intrinsic LEAK decreased by wk 8 (*p* = 0.01) and tended to decrease further from wk 8 to 12 (*p* = 0.08; Figures 6A,F). A trend for an age × training interaction suggested that while intrinsic LEAK decreased in young horses by wk 8 (*p* = 0.002) and remained lower at wk 12 than 0 (*p* = 0.0002), it only tended to decrease in aged horses from wk 0 to 12 (*p* = 0.06). Owing to these differences in adaptation, intrinsic LEAK was greater in aged than young horses at wk 8 (*p* = 0.03; Figures 6A,F). Throughout the study intrinsic P_CI_, E_CI+II_, and E_CII_ were greater in aged than young horses (Figure 6; *p* ≤ 0.05). A trend for an effect of training indicated that intrinsic P_CI_ (*p* = 0.06; Figures 6B,G) and E_CI+II_ (*p* = 0.10; Figures 6D,I) increased by wk 8 (*p* ≤ 0.04), but P_CI_ decreased from wk 8 to 12 (*p* = 0.05), and neither P_CI_ nor E_CI+II_ was different between wk 0 and 12. Intrinsic P_CI+II_ was greater in aged than young horses (*p* = 0.05; Figures 6C,H), but did not change with training. None of the measures of intrinsic oxidative capacity differed between muscle groups.

#### Flux Control Ratio

##### Age and Training

**FIGURE 7:**
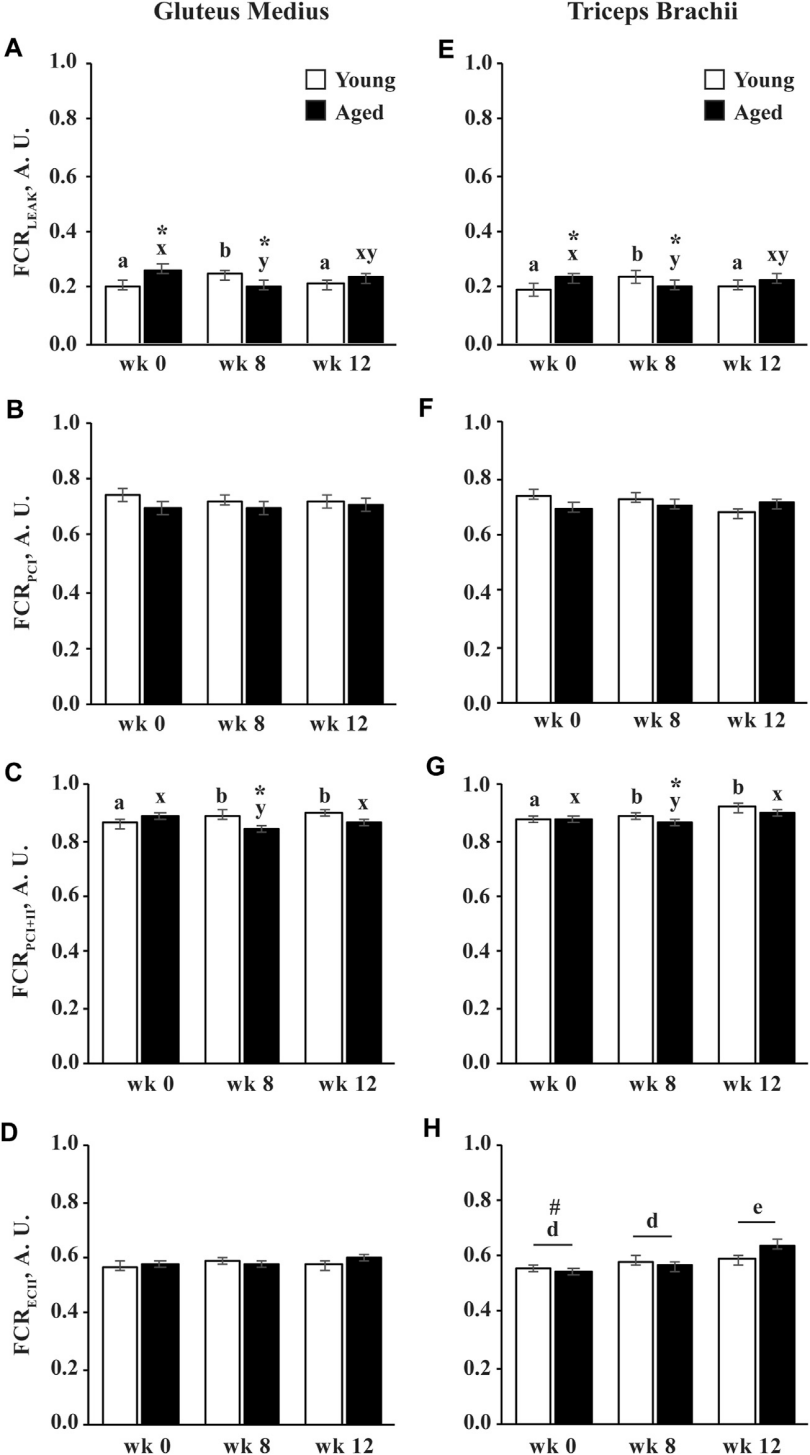
Flux control ratio (FCR) of permeabilized fibers from the gluteus medius and triceps brachii before (wk 0), and after 8 (wk 8) and 12 (wk 12) weeks of submaximal exercise training in aged (22 ± 4.5 yr; *n* = 10) and young (9.7 ± 0.7 mo; *n* = 8) horses. Contribution of LEAK respiration (LEAK; **A**,**E**), complex I-supported oxidative phosphorylation capacity (P_CI_; **B**,**F**), complex I and II-supported P (P_CI+II_; **C**,**G**), and complex II-supported electron transfer capacity (E_CII_; **D**,**H**) to maximal noncoupled E (E_CI+II_). Overall effect of age (*p* = 0.15; *p* = 0.05; *p* = 0.05; *p* = 0.04; *p* = 0.04), training (*p* = 0.0005; *p* = 0.056; *p* = 0.12; *p* = 0.10; *p* = 0.12), muscle group (*p* = 0.2; *p* = 0.5; *p* = 0.5; *p* = 0.3; *p* = 0.3), age × training (*p* = 0.06; *p* = 0.11; *p* = 0.2; *p* = 0.14; *p* = 0.2), age × muscle group (*p* = 0.7; *p* = 0.9; *p* = 0.99; *p* = 0.9; *p* = 0.8), training × muscle group (*p* = 0.3; *p* = 0.6; *p* = 0.5; *p* = 0.5; *p* = 0.4), and age × training × muscle group (*p* = 0.8; *p* = 0.9; *p* = 0.9; *p* = 0.9; *p* = 0.97) for FCR_LEAK_, FCR_PCI_, FCR_PCI+II_, and FCR_ECII_, respectively. * Within week, aged differs from young (*p* < 0.05). ^#^ Within week, GM differs from TB (*p* < 0.05). ^a,b^ Within young horses, time points with different letters differ (*p* < 0.05). ^x,y^ Within aged horses, time points with different letters differ (*p* < 0.05). ^d,e^ Within muscle group, time points with different letters differ (*p* < 0.05).

The flux control ratio (FCR) of each respiratory state is normalized to E_CI+II_, and therefore represents each state’s contribution to maximal noncoupled ETS capacity. The FCR for LEAK (FCR_LEAK_) was greater in aged than young horses before training (*p* = 0.01; Figures 7A,E). The FCR_LEAK_ increased in young horses (*p* = 0.009) and decreased in aged horses (*p* = 0.02) at wk 8 leading to a higher FCR_LEAK_ in young compared to aged horses at wk 8 (*p* = 0.03). The FCR_LEAK_ decreased in young horses from wk 8 to 12 (*p* = 0.03) and was not different between age groups at wk 12 (Figures 7A,E). The FCR for P_CI_ was not different between age groups and was not affected by training (Figures 7B,F). Similar to FCR_LEAK_, the FCR for P_CI+II_ increased in young horses (*p* = 0.02) but decreased in aged horses (*p* = 0.04) at wk 8 (Figures 7C,G). The FCR_PCI+II_ remained greater at wk 12 than wk 0 in young horses (*p* = 0.0006) and increased from wk 8 to 12 in aged horses (*p* = 0.02). By wk 12, the FCR_PCI+II_ was not different from wk 0 in aged horses and tended to remain lower than young horses (*p* = 0.08; Figure 7C). A trend for an interaction of age and training (*p* = 0.06) suggested that the FCR for E_CII_ increased in aged horses following 12 wk of exercise training (*p* = 0.004) and was greater in aged than young horses at wk 12 (*p* = 0.03; Figure 7D).

##### Muscle Group

The FCR for LEAK, P_CI_, and P_CI+II_ did not differ between muscle groups. The FCR_ECII_ was greater in the GM than the TB before exercise training (*p* = 0.03), but did not change in response to exercise training in the GM (Figure 7D). The FCR_ECII_ increased in the TB (*p* = 0.0004) following 12 wk of exercise training and was not different between muscle groups at wk 12 (Figure 7H).

## Discussion

In this study, we examined 10 aged and eight young horses to characterize the effects of age and 12 wk exercise training on skeletal muscle fiber type, satellite cell abundance, and mitochondrial density, function, and oxidative capacity. Before exercise training began, aged horses exhibited a lower percentage of IIx fibers and higher percentage of hybrid type IIa/x fibers, lower indices of satellite cell abundance, lesser mitochondrial function (CCO activity) and integrative oxidative capacity, and greater intrinsic oxidative capacity than young horses. Exercise training led to opposite effects on type IIx and IIa/x fiber percentages for aged compared to young horses, an increase in satellite cell abundance in aged horses, and an increase in integrative oxidative capacity for both the age groups. Thus, following 12 wk of light exercise training, aged horses had a higher percentage of IIx fibers and lower percentage of IIa/x fibers, greater satellite cell abundance, and similar integrative oxidative capacity compared to young horses, but sustained lower CCO activity and greater intrinsic oxidative capacity.

Before exercise training, aged horses exhibited a lower percentage of type IIx fibers in the GM compared to young horses, which is similar to previous research showing a lower percentage of MyHC IIx in aged compared to young horses (by western blotting) (Li et al., 2016) and humans (Verdijk et al., 2007). A reduction in percentage and size of type II fibers in aged individuals has been associated with decreased satellite cell abundance around type II fibers, and therefore a reduced ability to regenerate damaged fibers (Verdijk et al., 2007), which is further supported by lower satellite cell abundance around type II fibers observed in aged horses before training in the present study. Future research in horses and humans should verify changes in satellite cell content around type IIx versus type IIa fibers to solidify the causal relationship between loss of satellite cell abundance and atrophy.

Loss of type IIx fibers contributes greatly to loss of muscle mass and contractile function associated with sarcopenia during aging because type IIx fibers typically have the largest cross-sectional area and the fastest twitch speed. In addition to having a lower percentage of type IIx fibers, aged horses in the present study had a greater percentage of type IIa/x fibers than young horses in the GM before training. An increase in the percentage of hybrid fibers in aged subjects has been demonstrated in humans (Deschenes, 2004). An increase in the presence of hybrid fibers has been noted in both aging and disuse atrophy (Canepari et al., 2010), and may represent damaged muscle fibers (Adams, 2006). Therefore, the decrease in percentage of hybrid muscle fibers along with increases in satellite cell abundance and oxidative capacity in the present study may indicate an improvement in overall muscle health.

Although aged horses exhibited a lower percentage of type IIx fibers and a higher percentage of type IIa/x fibers when compared to young horses before training, the percentage of type IIx fibers in the GM increased, and the percentage of type IIa/x fibers in the GM decreased by wk 8 of exercise training in aged horses. These results are in agreement with some human literature indicating that resistance training results in a decrease in the percentage of hybrid fibers (Williamson, 2001). However, it is interesting to note that young horses exhibited the opposite adaptation, increasing in the percentage of hybrid type IIa/x fibers and decreasing the percentage of type IIx fibers, a result that has been previously reported in young horses entering an exercise training program (Revold et al., 2010). Research in humans (Williamson, 2001) and horses (Essèn-Gustavsson and Lindholm, 1985) typically demonstrates an increased percentage of type IIa fibers and a decrease in the percentage of type IIx fibers with training, regardless of intensity, which indicates a shift to a more oxidative muscle phenotype to support exercise activity. It is possible that the percentage of type IIx fibers increased in aged horses with training in the present study because they began with a decrement in type IIx fibers compared to young horses. Furthermore, all horses used in the present study were Quarter Horses, which have been shown to have a higher percentage of type IIx fibers compared to other breeds, such as Standardbreds (Bechtel and Kline, 1987). Therefore, the increase in the percentage of type IIx fibers with training in aged horses in the present study may not precisely represent the changes with training in aged horses of all breeds. Furthermore, the increase in percentage of nonoxidative fibers in the GM of aged horses was accompanied by similar oxidative capacity and mitochondrial volume density compared to young horses, suggesting that a shift to a more oxidative muscle fiber phenotype was not necessary to produce similar mitochondrial capacity in these horses.

In the present study, the size of type IIa fibers was larger in aged horses, and the size of type IIx fibers tended to be larger in aged compared to young horses. This is in contrast to literature in humans that shows a reduction in type II fiber size with aging (Deschenes, 2004). Greater fiber size in aged horses in the present study likely reflects the fact that young horses were still growing and had not reached their adult muscle fiber size. Therefore, comparisons of fiber size between aged and young horses in the present study may not fully reflect the change that may be observed from mature to aged groups. Interestingly, muscle fiber size did not increase in response to exercise training for either age group. This is in contrast to some human literature in aged populations showing that aerobic exercise commonly results in an increase in muscle fiber cross-sectional area after exercise training (Harber et al., 2009; Harber et al., 2012). However, it is similar to other literature in growing and mature horses (Yamano et al., 2002; Chanda et al., 2016) and elderly humans (Jubrias et al., 2001) undergoing long exercise training programs (16 wk–1 yr durations) that show no appreciable increase in muscle fiber size with exercise. The difference between studies likely results from a difference in exercise intensity. Similar to previous studies of elderly humans undergoing light exercise training (Jubrias et al., 2001), aged horses in the present study showed appreciable metabolic adaptations despite a lack of fiber hypertrophy.

Citrate synthase activity was measured as a marker of mitochondrial volume density (Larsen et al., 2012; Meinild Lundby et al., 2018). Aged horses in the present study exhibited higher CS activity in the TB than young horses. These results are contradictory to previous research that has shown lower CS activity in the TB of aged horses compared to young horses (Li et al., 2016). Citrate synthase activity has been shown to increase during the first year of life in horses (Kline and Bechtel, 1990). In the present study, young horses had a mean age of 9.7 mo, whereas in the aforementioned study, the young horses were older (1.8 ± 0.1 yr). Therefore, it is possible that mitochondrial density was still increasing in the young horses during the present study, masking differences that would typically be seen in comparison with more mature horses. Indeed, a limitation of this experiment is that the young horses used were still growing. This makes comparisons of CS activity and muscle fiber type and size difficult, because it is likely that the young horses in the present study had not reached their mature oxidative capacity and fiber size. Future research employing a wider range of ages of animals, or a more mature cohort of “young” animals to compare could provide insights into changes in these parameters over a wider range of the lifespan, and better inform adaptations to aging. That being said, the age of the young cohort of horses used in this study is representative of the age at which horses typically enter exercise training in the equine performance industry, and is, therefore, highly applicable and relevant.

Despite having higher mitochondrial volume density in the TB, aged horses had lower CCO activity, a marker of mitochondrial function on both an integrative and intrinsic basis. Decreased CCO activity with age has been well documented in humans (Müller-Höcker, 1990; Rooyackers et al., 1996) and more recently in horses (Li et al., 2016). Integrative CCO activity increased by wk 8 in both age groups, in agreement with previous studies showing improvements in CCO activity with exercise training in humans (Lanza and Nair, 2009), but integrative and intrinsic CCO activity remained lower for aged than young horses overall. Thus, while 12 wk light exercise training improved several measures of skeletal muscle mitochondrial health in aged horses, some measures remained depressed compared to young horses. To determine whether mitochondrial health in aged animals can be fully rescued, future research should focus on different lengths or intensities of exercise training, or combinations with pharmaceutical interventions aimed at improving mitochondrial health.

Integrative measures represent oxidative capacity of the muscle as a whole, without accounting for differences or changes in mitochondrial volume density. While integrative P_CI_ and E_CI+II_ were lower in aged than young horses before exercise training, they increased with training in aged horses, bridging the gap in capacity between age groups by wk 12. In this respect, exercise seems to have aided in correcting the deficiency in mitochondrial capacity for aged horses for these complexes. However, aged horses failed to match young horses’ adaptation to exercise training for maximal oxidative capacity (P_CI+II_). Additionally, the FCR for P_CI+II_, which is an indicator of efficiency of ATP production, was comparable between age groups at wk 0, but was lower in aged than young horses at wk 8 and tended to remain lower at wk 12. These differences between age groups arose because of an increase in the FCR for P_CI+II_ at wk 8 in young horses, but a decrease in aged horses at wk 8 and an increase at wk 12. The latency in the ability to improve efficiency of ATP production with training in aged horses suggests that aged horses may take longer to adapt metabolically to exercise training to meet energy demands.

A decrease in LEAK respiration has been demonstrated with exercise training in humans (Fernström et al., 2004), and the lag in decrease in intrinsic LEAK respiration in the present study indicates latency to improve mitochondrial efficiency with exercise training in aged horses, similar to the FCR_PCI+II_. However, LEAK respiration is a dissipative component for respiration associated with proton slip and leak and electron leak (Gnaiger, 2020). LEAK respiration is therefore not available for performing biochemical work, but has been suggested to be helpful in mitigating reactive oxygen species damage, which has been shown to increase with age (Brand, 2000). Further research is necessary to determine whether aged horses exhibit elevated reactive oxygen species production and oxidative stress in conjunction with increased LEAK respiration, and to determine whether decreasing oxidative stress facilitates a concomitant reduction in LEAK and improvement in mitochondrial efficiency.

Intrinsic oxidative capacity is normalized to muscle tissue CS activity, and therefore represents oxidative capacity normalized to mitochondrial density, which can be conceptualized as oxidative capacity “per mitochondria.” Overall, intrinsic P_CI_, P_CI+II_, and E_CI+II_ were greater in aged than young horses, suggesting that while aged horses may show decrements in oxidative capacity on a whole muscle scale, their capacity per mitochondria is actually elevated, perhaps as a compensatory mechanism. These results are similar to previous research in aged horses indicating elevated intrinsic P_CI_ and E_CI+II_ in aged horses (Li et al., 2016). However, in the previous study, integrative mitochondrial capacity was not different between age groups, whereas in the present study integrative P_CI_ and E_CI+II_ were lower in aged than young horses. In the previous study, the authors noted that they may not have observed decrements in integrative or intrinsic oxidative capacity because the horses may not have reached the age at which mitochondrial capacity becomes impaired, but instead horses may have been in more of a transitionary state (Li et al., 2016). The differences in integrative P_CI_ and E_CI+II_ may have arisen because the aged horses in the present study were older (18–30 yr) than the horses in the previous study (17–25 yr) and may have had more advanced decrements in mitochondrial capacity due to more advanced age.

Furthermore, aged horses showed differing changes on the reliance of complexes I and II over time in response to exercise training. In the present study, intrinsic P_CI_ increased from wk 0 to 8 but tended to decrease from wk 8 to 12. The decrease in intrinsic P_CI_ at wk 12 may result from a shift to reliance on complex II during adaptation to training. While the FCR for P_CI_ did not change in response to training in either age group, the FCR for P_CI+II_ increased at wk 8 and remained higher at wk 12 in young horses and the FCR for P_CI+II_ decreased at wk 8 in aged horses and increased at wk 12. Complex II links the tricarboxylic acid cycle to the ETS, and therefore influences the rate of substrate entry into the ETS. Increasing capacity for substrate entry into the ETS contributes to increasing reserve respiratory capacity, which is important in cases where energy demands exceed supply (Pfleger et al., 2015). Therefore, an increase in complex II capacity and preference with exercise results in the ability to avoid “ATP crisis” during exercise bouts. The increase in FCR for E_CII_ coupled with the lack of change in complex I reliance suggests that aged horses may increase reliance on complex II with exercise training to adapt to the energy demands of exercise. Additionally, complex I capacity has been associated with increased reactive oxygen species production (St-Pierre et al., 2002). Thus, a decrease in reliance on complex I activity may be favorable, particularly in aged horses, if they exhibit similar age-associated increases in oxidative stress to other species (Brand, 2000).

The present study demonstrates deficits in mitochondrial function and oxidative capacity and alters fiber type in aged compared to young horses that are similar to the deficits and differences observed in aged humans. Exercise training in both the age groups resulted in improvements in many indicators of mitochondrial function and density and resulted in a decrease in percentage of hybrid fibers in aged horses, as has been demonstrated in humans. Taken together, these results suggest that exercise could be an extremely useful tool for improving the health and welfare of aged horses. Additionally, the phenotypic similarities of aged horses and aged humans show that aged horses can be a useful model for aging in human skeletal muscle.

## Data Availability

The raw data supporting the conclusions of this article will be made available by the authors, without undue reservation.

## Supplementary Material

Supplementary Figure 1 Example output from a high-resolution respirometry (HRR) experiment on the Oxygraph-2k using permeabilized skeletal muscle fibers. O2 concentration (blue line) is on the left y-axis and O2 flux per mass (pmol/sec/mg tissue) is on the right y-axis, with time on the x-axis. The SUIT protocol was as follows: (1) pyruvate (5 mM) and malate (1 mM) to support electron flow through complex I (CI) of the electron transfer system (ETS; LEAK respiration); 2) adenosine diphosphate (ADP; 2.5 mM) to stimulate respiration (OXPHOS) and cytochrome c (cyt c; 10 μM) to assess outer mitochondrial membrane integrity; 3) glutamate (10 mM) as an additional CI substrate to assess OXPHOS with complex I (PCI); 4) succinate (10 mM) to support convergent electron flow through complex II (CII) of the ETS (PCI+II); 5) uncoupler carbonyl cyanide 3-chlorophenylhydrazone (CCCP; 0.5 μM steps) to assess maximal noncoupled ETS capacity (ECI+II); 6) rotenone (0.5 μM), an inhibitor of complex I, to measure ETS capacity of complex II alone (ECII); 7) antimycin A (2.5 μM), an inhibitor of complex III, to measure residual oxygen flux (ROX) independent of the ETS.

## Abbreviations

CCO, Cytochrome *c* oxidase activity; CI, Complex I; CII, Complex II; CS, Citrate synthase activity; E, Electron transfer system capacity; FCR, Flux control ratio; GM, Gluteus medius; MyHC, Myosin heavy chain; P, Oxidative phosphorylation capacity; TB, Triceps brachii.
