# Supplementary figures and images for: Skeletal Muscle Adaptations to Exercise Training in Young and Aged Horses
Source: Front Aging. 2021 Oct 27;2:708918. doi: 10.3389/fragi.2021.708918 (PMC9261331; doi:10.3389/fragi.2021.708918)

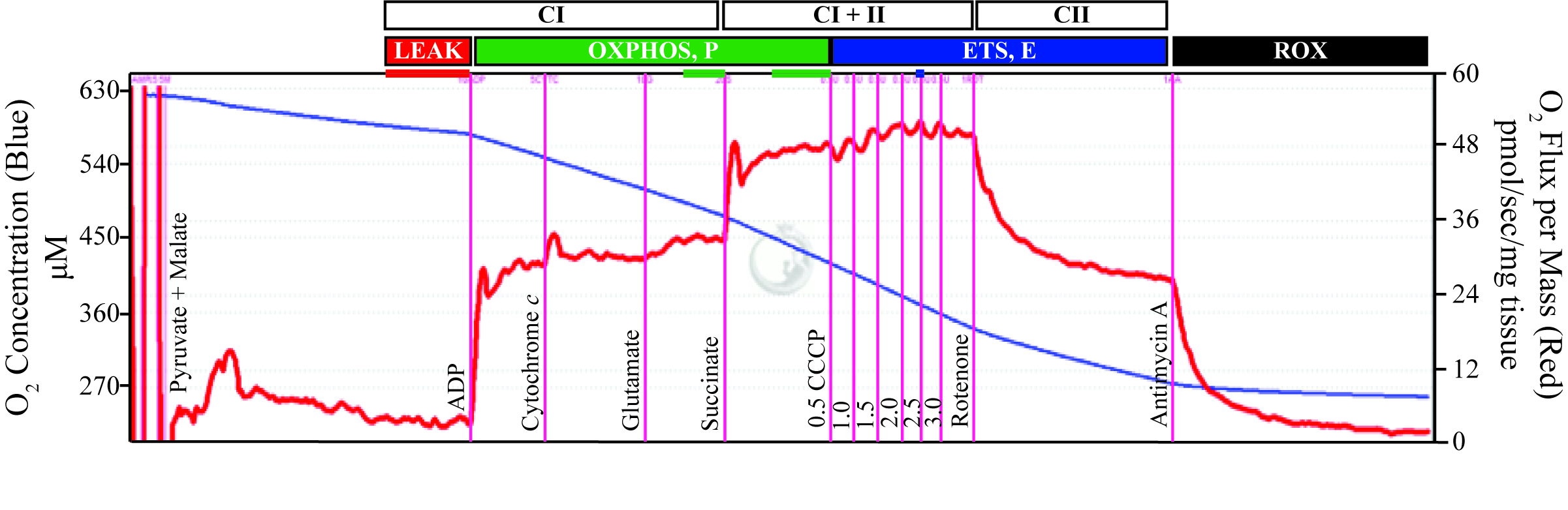

Supplement: Supplementary file 1 [file Image1.TIF]
